# Nanopore sequencing as a novel approach to transcend into the deep universe of schizophrenia

**DOI:** 10.1192/j.eurpsy.2023.944

**Published:** 2023-07-19

**Authors:** I. B. Nita, O. D. Ilie, A. S. Ciobica, L. D. Hritcu, R. Popescu, R. P. Dobrin

**Affiliations:** 1 Department of Medicine III, “Grigore T. Popa” University of Medicine and Pharmacy; 2 Department of Biology, “Alexandru Ioan Cuza” University; 3 Internal Medicine Clinic, “Ion Ionescu de la Brad” University of Life Sciences; 4 Department of Medical Genetics, “Grigore T. Popa” University of Medicine and Pharmacy, Iasi, Romania

## Abstract

**Introduction:**

Schizophrenia (SCZ) is a chronic neuropsychiatric disorder possessing a multifactorial nature and dual facets of symptoms with a core underlying genetic mechanism that is still obscure. Lately, genomic studies revealed numerous single nucleotide polymorphisms (SNPs) that are non-coding and influence ribonucleic acid (RNA) expression, particularly its splicing.

**Objectives:**

Considering that next-generation sequencing (NGS) protocols focus upon long-read sequencing as opposed to conventional RNA sequencing methodologies once with the advent of Oxford Nanopore Technologies’ (ONT) MinION, we primarily aimed to gather and review all evidence into how this approach may deepen and further offer insight into SCZ still undiscovered domain.

**Methods:**

The relevant literature searches were performed using distinct combinations of keywords including “schizophrenia” alongside „Nanopore”, “MinION”, and “Oxford Nanopore Technologies” on four databases (PubMed/Medline, ISI Web of Knowledge, Scopus, and ScienceDirect). We implied the entries to strictly “research articles” written in English as inclusion criteria.

**Results:**

By restricting the returned results starting with the year when the platform was officially launched, a total of n = 69 studies were displayed between the pre-established interval (2014 – 2022). If taken per database, n = 2 were identified in PubMed/Medline, n = 7 in ISI Web of Knowledge, n = 4 in Scopus, and n = 56 in ScienceDirect. In chronological order, n = 0 were published in 2014, n = 3 in 2015, n = 7 in 2016, n = 7 in 2017, n = 9 in 2018, n = 3 in 2019, n = 7 in 2020, n = 19 in 2021 and n = 14 in 2022. Finally, per the strategy applied, n = 49 were returned for “schizophrenia” + “Nanopore” from which n = 2 in PubMed/Medline, n = 5 in ISI Web of Knowledge, n = 4 in Scopus, and n = 38 in ScienceDirect. For “schizophrenia” + “MinION, there was a cumulative number of n = 5, from which we had n = 0 in PubMed/Medline, n = 0 in ISI Web of Knowledge, n = 0 in Scopus, and n = 5 in ScienceDirect. Finally, for “schizophrenia” + “Oxford Nanopore Technologies” were displayed n = 15, and the situation was n = 0 in PubMed/Medline, n = 2 in ISI Web of Knowledge, n = 0 in Scopus, and n = 13 in ScienceDirect.

**Conclusions:**

We presently assist to a fulminant ascension in the literature, with applicability in other fields. Perhaps as cornerstone stands a recent publication in which the authors reveal the risk of the Calcium Voltage-Gated Channel Subunit Alpha1 C (*CACNA1C*) gene involved, being identified thirty-eight novel exons and two hundred and forty-one novel transcripts following RNA purification from six regions (cerebellum, striatum, and dorsolateral prefrontal cortex) among which targeted were cingulate, occipital and parietal cortexes.

**Disclosure of Interest:**

None Declared

